# COVID-19: Vaccination Side Effects and Sick Leave in Frontline Healthcare-Workers—A Web-Based Survey in Germany

**DOI:** 10.3390/vaccines10030411

**Published:** 2022-03-09

**Authors:** André Nohl, Bastian Brune, Veronika Weichert, Fabian Standl, Andreas Stang, Marcel Dudda

**Affiliations:** 1 Emergency Medical Services, Fire Brigade Oberhausen, 46047 Oberhausen, Germany; 2 Department of Emergency Medicine, BG Klinikum Duisburg, 47249 Duisburg, Germany; 3 Department of Trauma, Hand and Reconstructive Surgery, University Hospital Essen, 45147 Essen, Germany; marcel.dudda@uk-essen.de; 4 Helicopter Emergency Medical Service (HEMS), 47249 Duisburg, Germany; veronika.weichert@bg-klinikum-duisburg.de; 5 Emergency Medical Services, Fire Brigade Essen, 45139 Essen, Germany; 6 Department of Trauma and Reconstructive Surgery, BG Klinikum Duisburg, 47249 Duisburg, Germany; 7 Institute of Medical Informatics, Biometry, and Epidemiology, University Hospital Essen, 45147 Essen, Germany; fabian.standl@uk-essen.de (F.S.); imbe.dir@uk-essen.de (A.S.); 8 Department of Epidemiology, School of Public Health, Boston University, Boston, MA 02215, USA

**Keywords:** COVID-19, SARS-CoV-2, vaccination, side effects, EMS, frontline health care workers

## Abstract

(1) Background: The COVID-19 vaccination has caused uncertainty among employees and employers regarding vaccination reactions and incapacitation. At the time of our study, three vaccines are licensed in Germany to combat the COVID-19 pandemic (BioNTech/Pfizer (Comirnaty), AstraZeneca (Vaxzevria), and Moderna (Spikevax). We aim to assess how often and to what extent frontline healthcare workers had vaccination reactions after the first and second vaccination. The main focus is on the amount of sick leave after the vaccinations. (2) Methods: We create a web-based online questionnaire and deliver it to 270 medical directors in emergency medical services all over Germany. They are asked to make the questionnaire public to employees in their area of responsibility. To assess the association between independent variables and adverse effects of vaccination, we use log-binomial regression to estimate prevalence ratios (PR) with 95% confidence intervals (95%CI) for dichotomous outcomes (sick leave). (3) Results: A total of 3909 individuals participate in the survey for the first vaccination, of whom 3657 (94%) also provide data on the second vaccination. Compared to the first vaccination, mRNA-related vaccine reactions are more intense after the second vaccination, while vaccination reactions are less intense for vector vaccines. (4) Conclusion: Most vaccination reactions are physiological (local or systemic). Our results can help to anticipate the extent to which personnel will be unable to work after vaccination. Even among vaccinated HCWs, there seems to be some skepticism about future vaccinations. Therefore, continuous education and training should be provided to all professionals, especially regarding vaccination boosters. Our results contribute to a better understanding and can therefore support the control of the pandemic.

## 1. Introduction

### 1.1. COVID-19 Pandemic

In 2019, a novel coronavirus was identified in Wuhan, China. The virus was referred to as “severe acute respiratory syndrome coronavirus 2” (SARS-CoV-2) because of the respiratory symptoms that were first recognized. The WHO named the associated disease COVID-19 (Coronavirus Disease 2019) [1,2,3]. The COVID-19 virus led to a global pandemic and a particular challenge for the health system [4]. The clinical picture varies from asymptomatic to life-threatening courses with acute pulmonary and multiple organ failure as well as thromboembolic events. Older people and people with serious pre-existing illnesses are more likely to have complicated or fatal courses [5,6,7].

The SARS-CoV-2 pandemic and the different manifestations of the disease COVID-19 are a major challenge to health systems worldwide. The pandemic caused massive restrictions on the population with different regional dimensions. In several hotspots, there were temporary capacity shortages in hospitals and personnel. Frontline healthcare workers (HCW) play a special role in containing the pandemic.

### 1.2. Infection Risk for HCW’s and Patients

SARS-CoV-2 shows a high level of infectivity and, compared to influenza viruses, a longer survival time on surfaces, so that a particular risk to HCW can be assumed [8,9]. However, the greatest risk of virus transmission is through droplets and aerosols [10,11,12]. To avoid patient-to-staff and staff-to-patient transmission, adherence to strict hygiene standards is important and crucial to maintaining a maximum workforce [13,14]. Because of procedures that cannot be planned, such as those required in the context of resuscitation measures or in airway management, frontline HCWs have a higher risk than personnel without contact with patients or emergency procedures. On the one hand, they have a higher risk of infecting themselves, and on the other hand, there is also an increased risk of infecting patients due to close contact [5,15,16]. 

Lapolla et al. [17] published 206 deaths of frontline HCWs in Italy, the initially most affected region, by 16 April 2020. About 43.3% were aged 60–69 years and 26.7% were aged 50–59 years. HCWs aged 70–79 years comprised 20% of HCW deaths. Hence, medical personnel with regular direct contact with patients have been classified in category 1 of the vaccination order.

### 1.3. Vaccination Strategy in Germany

The early vaccination strategy in Germany was divided into several phases. At the beginning of vaccine licensing and the corresponding vaccine shortage, a vaccination strategy with several priorities was established in Germany. The highest priority was given to persons at high risk for severe courses of infection by COVID-19 (e.g., advanced age > 80 years, severely pre-diseased patients). Subsequent priority was given to frontline HCWs [18].

In the German vaccine distribution strategy, two vaccines, Comirnaty (BioNTech Manufacturing GmbH) and Spikevax (formerly COVID-19 vaccine Moderna, Moderna Biotech Spain, S. L.) were initially provided for hospitals and EMS with an offset of a few weeks. Vaxzevria (AstraZeneca AB) was approved later after a large part of the frontline HCWs had already been vaccinated. All vaccines were approved by the European Medicines Agency (EMA) for Germany and the European Union (conditional marketing authorization (CMA)).

### 1.4. Vaccination Side Effects

Typical symptoms after vaccination in general are erythema, swelling, and pain at the vaccination site. However, general symptoms such as fever, joint pain, and headache may also occur. These reactions are desired responses of the immune system and usually subside after a few days. Information on vaccination reactions can usually be found in the vaccine’s respective technical information [19,20].

So-called “serious adverse vaccine reactions” after vaccinations are rare. If an adverse drug reaction is suspected or confirmed, there is a legal obligation in Germany to report these complications to the municipal health department (§6.1 German Infection Protection Act). The department forwards these cases to the competent state authority, the competent higher federal authority, and the Paul Ehrlich Institute.

### 1.5. Aim of the Study

The aim of our study is to analyze the frontline HCW’s vaccination reactions and side effects to the three approved vaccines.

With this study, we would like to evaluate the extent to which sick leave can be expected after COVID-19 vaccinations. Healthcare personnel are already under a great burden due to the pandemic. In addition, a further burden occurs due to the sick leave of urgently needed personnel after the very important COVID-19 vaccination. The evaluation of sick leaves could be useful to develop a vaccination strategy in the future without causing further staff shortages.

## 2. Materials and Methods

### 2.1. Study Design

We conducted a web-based online survey (Umfrageonline.com (last accessed 10 October 2021, enuvo, Zurich, Switzerland). Participation was possible from 23 February 2021 until 29 March 2021.

### 2.2. Target Population

Our primary target population were frontline HCWs and therefore we primarily addressed emergency medical services (EMS) personnel through their medical directors. We contacted 270 of 401 medical directors of EMS in cities and counties in Germany. Contact data for the remaining 131 medical directors were unavailable. We asked all medical directors to forward the survey link and a flyer with QR code to the rescue stations. On 31 March 2021, 73,333 employees were subject to social insurance contributions in the emergency medical services sector in Germany [21].

Since many medical directors work in both COVID-19 crisis teams and hospitals, it is possible for them to get the survey out to hospitals and nursing facilities as well. Therefore, personnel from hospitals had the opportunity to participate in our survey as well. Often physicians from hospitals also work in EMS. Depending on their qualifications, paramedics and nurses can also work in both hospitals and EMS. In order to consider activity in more than one area, it was possible to select more than one answer option.

In the German EMS system, prehospital emergency care is primarily provided by EMS personnel, supported by an on-scene emergency physician in life-threatening situations. The competence of EMS personnel includes a range of Advanced Life Support (ALS) treatments, which EMS personnel must perform until an emergency physician arrives on the scene. At that point, paramedics (and other medical personnel on scene) act under the direct medical supervision of the physician [22]. Other (non-life-threatening) emergencies are treated independently by the EMS personnel without a physician being on site. EMS personnel have direct contact with all patient groups and are therefore classified as frontline HCWs [17].

### 2.3. Questionnaire

Questions referring vaccines, demographic data (sex, federal state), profession, number of contacts with COVID-19 cases, and their own medical history were formulated closed as multiple-choice questions (Q1, 3–11, 24, 25, 27, 28). Answers about age and sick leave were given in free text fields (Q2, 22, 23). In each case for the 1st and 2nd vaccination, the information on the administered vaccine was given. The medical history included information on previous diseases and long-term medication use. They were standardized in an Excel table and checked for plausibility in each case. To assess the extent of side effects and attitudes toward future vaccinations, questions on these topics were answered on visual analog scales (one-point steps from 0 = not applicable to 100 = fully applicable; Q12–21, 26, 29). The following side effects were documented: local, systemic, neurological, allergic, and other. The questionnaire was developed by the core study group (medical directors and experts in public health and statistics) and tested by 5 medical directors in emergency medical services. Proposed changes have been adopted.

### 2.4. Statistical Methods

We express values of the visual analog scale (0–100) as percentages, e.g., a value of 31 is reported as 31%. We report absolute counts and percentages for categorical data. For continuous data, we report means and standard deviations or medians and interquartile ranges where appropriate. To assess the association between independent variables and adverse effects of vaccination we used log-binomial regression to estimate crude and adjusted (age, sex, and profession) risk ratios (RR) with 95% confidence intervals (95%CI) for the occurrence of sick leave.

We are calculating and reporting confidence intervals to assess the precision of our estimates because our goal is estimation and not significance testing. We wish to avoid publication bias by preferential reporting of significant results. Instead, we judge the value of our estimates by their precision and validity [23,24].

Statistical analyzes were performed using IBM^®^ SPSS^®^ Statistics Version 27.0 (IBM Corporation, Armonk, NY, USA) and SAS 9.4 (Cary, NC, USA).

### 2.5. Ethical Consideration

Participants were informed at the beginning of the survey that participation was voluntary and anonymous. They were also informed that the results would be analyzed and published. Participation could be interrupted at any time. The study is consistent with the Declaration of Helsinki. The ethics committee of the University of Duisburg-Essen has approved our study (21-9929-BO).

## 3. Results

Of the 4620 people who opened the online questionnaire, 711 people thereafter decided not to fill-in the questionnaire and had to be excluded. Another four people were excluded because they did not report the type of vaccination, leaving 3905 people for the analysis of side effects after the first vaccination. Among these, 3657 (94%) also filled in the questionnaire after the second vaccination.

The median ages differed by the following professional groups: physicians and others (41 years), EMS personnel (34 years), and nurses (39 years). Age was not associated with sex (both median age of 38 years, diverse excluded) (Table 1). BioNTech/Pfizer (76.7%) was the most frequent vaccine, followed by AstraZeneca (15.8%) and Moderna (7.45%) (Table 2). A total of 48.3%, 51.5%, and 0.2% self-identified as male, female, and diverse, respectively.

The largest group of participants were emergency services personnel (39.4%), followed by nurses (36.5%) and physicians (12.7%). People vaccinated with BioNTech were slightly older than people vaccinated with other vaccines (median age, 38 years vs. 36 years). While EMS was dominated by men (78.7%), the nursing service and others were dominated by women (80.1% and 71.6%, respectively). Among physicians, men predominated only slightly (54.5% vs. 45.5%). While men made up 52% of the participants in BioNTech, this proportion was only 25% in Moderna.

The need for medication after the first and second vaccinations depended on the vaccine. While after the first vaccination with BioNTech, Moderna, and AstraZeneca in 10.0%, 23.0%, and 68.7%, respectively, this need was 37.6%, 70.0%, and 31.6%, respectively, after the second vaccination.

Among the reactions elicited (local, systemic, allergic, neurologic, and other reactions), increased VAS scores were observed essentially only for local and systemic reactions. For the first vaccination, local reactions were strongest for Moderna, while systemic reactions were strongest for AstraZeneca. At the second vaccination, the expression of local reactions for BioNTech and Moderna was nearly the same as at first vaccination, while significantly fewer local reactions were reported for AstraZeneca at second vaccination.

At the first vaccination, systemic reactions were much less for BioNTech and Moderna than for AstraZeneca, while at the second vaccination, systemic reactions were markedly more for BioNTech and Moderna than for AstraZeneca (Table 3).

After the first vaccination, the intensity of local and systemic vaccine reactions (VAS: visual analog scale) tended to be lower the older the age was for all types of vaccinations, with the following exception: for the systemic reaction after BioNTech vaccination, there was virtually no relationship between the intensity of systemic reactions and age at vaccination. Moreover, after the second vaccination, local, and systemic reactions tended to be lower the older the person was, with the exception of Moderna (Table 4).

Table 5 shows the number and percentage of sick days after the first and second COVID-19 vaccinations. Here it is shown that the number of sick days increases after the second vaccination.

Overall, 13.4% were unable to work for at least one day after the first vaccination. The percentage of HCWs who were unable to work for at least one day after the first vaccination differed by vaccine as follows: BioNTech 4.4%, Moderna 13.1%, and AstraZeneca 55.0%.

Compared with BioNTech, age-, sex-, and profession-adjusted sick leave after the first vaccination was 2.9 times more likely with Moderna (95%CI: 2.0–4.0) and 11.6 times more likely with AstraZeneca (95%CI: 9.7–14.0). In contrast, after the second vaccination, age-, sex-, and profession-adjusted sick leave was 2.0 times more likely with Moderna (95%CI: 1.8–2.3) and 0.7 times less likely with AstraZeneca (95%CI: 0.6–0.9) (Table 6).

Willingness for future vaccination of any kind showed a U-shaped distribution on the VAS scale with stronger occupations on the edges of the scale, resulting in large interquartile ranges. Vaccination propensity differed markedly depending on the COVID-19 vaccine. After the second vaccination, individuals vaccinated with BioNTech or Moderna had markedly stronger vaccination readiness (median and interquartile range [IQR]: BioNTech 89 [47–100], Moderna 92 [47–100]) than individuals vaccinated with AstraZeneca (56, [16–99]).

## 4. Discussion

Based on our results, we can conclude that especially local and systemic reactions occur after both the first and second vaccinations. However, the mRNA vaccines have less intense reactions after the first vaccination than after the second vaccination. In contrast, the local and systemic reactions are less pronounced in the AstraZeneca vector vaccine during the second vaccination. This difference between mRNA and vector vaccine is also reflected in the proportion of HCWs with sick leave for at least one day. After the second vaccination with the mRNA vaccines, sick leave was higher. In contrast, sick leave was lower after the second vaccination with the AstraZeneca vector vaccine compared to the first vaccination.

Vaccination reactions appear to be weaker the higher the age. The following results are also evident in older studies: B-cell responses are lower at older ages. Older people are also no longer able to respond quickly and persistently to new antigens [25]. However, a recent study shows that vaccine efficacy in subjects over 65 years of age was no lower than in younger subjects [26]. Otherwise, older persons may have a different awareness of health and disease due to increased life experience as well as experience with diseases. Accordingly, it could also be that the existing symptoms after vaccinations could be subjectively perceived as less.

Overall, local and systemic vaccine side effects predominated in our study and were similar to the results from Germany [27]. Serious adverse effects could not be detected with certainty in our study because the severity of symptoms was only documented by a visual analog scale and was self-reported. A distinction between the usual immune response (usual local and systemic reactions) and severe complications is therefore not possible. Our results are comparable to those of the Paul Ehrlich Institute. The Paul Ehrlich Institute reports 106,835 suspected cases. According to the Robert Koch Institute, over 74 million vaccinations were given by 30 June 2021, including over 54 million vaccinations with Comirnaty, over 6 million vaccinations with Spikevax, over 11 million vaccinations with Vaxzevria, and over 1 million vaccinations with the Janssen COVID-19 vaccine. 49,735 suspected cases were reported for vaccination with Comirnaty, 14,153 suspected cases were reported for Spikevax, 39,398 suspected cases were reported for Vaxzevria, and 3061 were reported for the COVID-19 vaccine by Janssen. In 488 reported suspected cases, the COVID-19 vaccine was not specified. The reporting rate was 1.4 per 1000 vaccine doses for all vaccines combined and 0.1 per 1000 vaccine doses total for reports of serious reactions [28].

Accordingly, in our collective, based on the PEI data, with a participant number of almost 4000 HCWs, a serious complication would have been expected in 0.8 participants (if all participants had received the second vaccination dose).

The CDC identified 66 case reports received by the vaccine adverse event reporting system (VAERS) that met Brighton Collaboration case definition criteria for anaphylaxis (levels 1, 2, or 3): 47 following the Pfizer-BioNTech vaccine, for a reporting rate of 4.7 cases/million doses administered, and 19 following the Moderna vaccine, for a reporting rate of 2.5 cases/million doses administered [29]. Twenty-one (32%) of the 66 case reports noted a prior episode of anaphylaxis from other exposures, including 18 in intensive care, 7 of whom required endotracheal intubation, and hospitalization ranged from one to three days.

Based on our results, it is reasonable to assume that a booster vaccination, especially with an mRNA vaccine, may lead to renewed service absences and should therefore be considered in the duty scheduling of personnel.

It is well known that in recent years, there has been an overall decrease in vaccination readiness in Europe. A recent European survey on the willingness of the population to be vaccinated against COVID-19 shows disappointing results. In turn, concern about possible side effects increased [30]. Several studies show a low willingness for seasonal influenza and COVID-19 vaccination among HCW and also EMS personnel. Reasons often given are the following: self-determination, sufficient health status, fear of adverse effects, and concerns about safety and efficacy [8,31]. Inadequate vaccination compliance was also reflected among EMS personnel [32,33]. An effect of the now available personal protective equipment, information about vaccination reactions, and side effects on the willing is possible.

In the case of the AstraZeneca vaccine, serious but rare complications associated with vaccination have been shown to occur in Germany and the EU. Vaccination was then paused for a short time. However, after further investigations, the vaccination was again approved by the European Medicines Agency [34,35,36]. Our study was conducted at the time of inconsistent reports about AstraZeneca. The uncertainty and bad news, such as side effects after the first vaccination, may have led HCWs to overestimate side effects and be more skeptical of future vaccinations in this group. However, the overall future vaccination willingness is still too low to achieve a safe herd immunity, according to our results. In particular, a recent study shows that vaccine efficiency decreases with decreasing antibody levels after the second COVID-19 vaccination with BioNTech and that a so-called booster vaccination will be necessary [37]. Thus, these results confirm our first study to survey the vaccination willingness of frontline HCWs before the start of the first vaccination [31]. Apparently, even medically educated people remain skeptical even though they have already been vaccinated. Therefore, a continuous information campaign will remain necessary, especially for these professional groups. Our results may help to show that mainly local and systemic side effects occurred, reflecting a physiological immune response.

Our study has strengths and limitations. We present one of the very first studies that has evaluated the inability to work in HCWs after COVID-19 vaccinations and can therefore help to better calculate employee absences in future vaccinations. Furthermore, our study used a visual analog scale to assess the intensity of adverse effects on a metric scale. Our sample, compared to similar studies, is larger than average. To our knowledge, this is the first study in the world that also evaluates HCWs’ work absence after COVID-19 vaccination.

Because of the study design, the reported symptoms and absences could not be validated. The questionnaire asked whether the patient had taken any medication after the vaccination. However, it was not considered whether medication was taken prophylactically to suppress any symptoms that might occur. In this case, a bias may occur, although the side effects are likely to have been milder. We also cannot differentiate whether the responses are typical vaccine reactions, adverse drug reactions, or vaccine injuries. No data were collected on the participants’ work experience. Work experience could influence the results. The following conditions during the survey could not be controlled: whether the participant was distracted, whether other people were present, influencing the processing, or whether some people participated more than once, it could not be traced. It might be possible that HCWs with side effects after vaccinations tended to participate more than HCWs without side effects.

Most vaccination reactions are physiological (local or systemic) and exist for a short time. Incapacity to work and vaccine reactions were more frequent after the second vaccination for mRNA vaccines. For vector vaccines, incapacity to work and vaccine reactions were less frequent after the second vaccination. Younger participants and women experienced increased sick days and vaccination reactions. The higher the medical education level, the less frequent are sick leaves and adverse reactions after vaccination.

## 5. Conclusions

Our results can help to anticipate the extent to which personnel will be unable to work after vaccination. Even among vaccinated HCWs, there seems to be some skepticism about future vaccinations. Therefore, continuous education and training should be provided to all professionals, especially regarding a booster vaccination. Corresponding absences of personnel would have to be planned accordingly. Our results contribute to a better understanding and can therefore better support the control of the pandemic.

## Figures and Tables

**Table 1:** Demographic data of 3905 frontline health care workers in Germany, 23 February–29 March 2021.

Demographic Data (*n* = 3905)		Frequency (*n*)	Percent (%)
Sex	Male	1886	48.3
	Female	2010	51.5
	Diverse	9	0.2
Professional group	Physicians	497	12.7
	EMS personnel	1538	39.4
	Nurses	1426	36.5
	Others	444	11.4
Field of work	EMS	1785	45.7
	Patient transport	618	15.8
	ICU	646	16.5
	General ward	668	17.1
	Emergency department	363	9.3
	Infection ward	395	10.1
	Nursing home	309	7.9
	Outpatient elderly care	205	5.2
	In training	225	5.8
	Others	720	18.4
Federal state	Baden-Württemberg	296	7.6
	Bavaria	391	10
	Berlin	59	1.5
	Brandenburg	83	2.1
	Bremen	8	0.2
	Hamburg	45	1.2
	Hesse	271	6.9
	Mecklenburg-Western Pomerania	34	0.9
	Lower Saxony	401	10.3
	North Rhine-Westphalia	1891	48.4
	Rhineland-Palatinate	120	3.1
	Saarland	29	0.7
	Saxony	70	1.8
	Saxony-Anhalt	81	2.1
	Schleswig-Holstein	87	2.2
	Thuringia	39	1
		Median (IQR)	mean (SD)
Age (years)	Male	38 (16)	38.6 (11.22)
	Female	38 (18)	38.9 (11.3)
	Divers	31 (24)	36.2 (12.1)
	Physicians	41 (16)	42.1 (10.41)
	EMS personnel	34 (18)	35.8 (10.96)
	Nurses	39 (18)	39.8 (10.96)
	Others	41 (21)	41.4 (11.84)
	BioNTech	38 (17)	38.9 (11.18)
	Moderna	36 (19)	38.1 (11.41)
	AstraZeneca	36 (19)	38 (11.58)

Legend: EMS emergency medical services, ICU intensive care unit, IQR interquartile rage, SD standard deviation.

**Table 2:** Descriptive data with medical content.

Descriptive Data (Medical Informations, *n* = 3905)		Frequency (*n*)	Percent (%)
No. of COVID-19 Patients Treated	0	772	19.8
	Up to 5	845	21.6
	Up to 10	573	14.7
	Up to 20	697	17.8
	Up to 50	632	16.2
	Up to 100	253	6.5
	Over 100	132	3.4
Relatives Infected	No	3496	89.5
	Yes	408	10.4
Self-Infected	No	3377	86.5
	I don’t know	377	9.7
	Yes without symptoms	127	3.3
	Yes with symptoms	23	0.6
Health Status of Participants			
Diseases	Healthy	2361	60.5
	Obesity	783	20.1
	Metabolic disease	176	4.5
	Immunodeficiency	66	1.7
	Respiratory disease	345	8.8
	Cardiovascular disease	428	11
	neurological disease	87	2.2
	Malignancies and tumors	35	0.9
	Other	266	6.8
Medications	None	2517	64.5
	Immunosuppressants	67	1.7
	Antidiabetics	79	2
	Anticoagulants	88	2.3
	Inhalatives	128	3.3
	Chemotherapeutics	9	0.2
	Analgesics	92	2.4
	Other	1109	28.4
Vaccine used	BioNTech	2997	76.7
	AstraZeneca	617	15.8
	Moderna	291	7.5

**Table 3:** Median values and interquartile ranges of the visual analog scale for local and systemic reactions depending on the vaccine.

Vaccine and Reaction	First Vaccination		Second Vaccination	
	Median	IQR	Median	IQR
Local reactions				
BioNTech	20	40	20	44
Moderna	48	53	51	49
AstraZeneca	30	59	3	39
Systemic reactions				
BioNTech	1	22	34	74
Moderna	12	40	79	50
AstraZeneca	72	66	11	54

**Table 4:** Association between age at first and second vaccination and local and systemic reactions as measured by a visual analog scale by type of vaccination.

Reaction	Vaccination	Number of People	Spearman Rank Correlation Coefficient	95% Confidence Interval
First vaccination				
Local reaction	Biontech	2992	−0.23	−0.26; −0.19
	Moderna	291	−0.25	−0.36; −0.14
	Astra	609	−0.22	−0.30; −0.14
				
Systemic reaction	Biontech	2988	−0.04	−0.08; 0.00
	Moderna	291	−0.11	−0.23; 0.00
	Astra	609	−0.27	−0.34; −0.20
Second vaccination				
Local reaction	Biontech	2966	−0.18	−0.21; −0.14
	Moderna	265	−0.10	−0.22; −0.02
	Astra	347	−0.23	−0.33; −0.13
Systemic reaction	Biontech	2968	−0.09	−0.13; −0.06
	Moderna	266	−0.01	−0.13; 0.11
	Astra	352	−0.23	−0.32; −0.12

Legend Table: 95% confidence intervals by Fisher’s z-transformation.

**Table 5:** Number and percentages of sick leave days after the first and second COVID-19 vaccination.

Number of Sick Leave Days	First Vaccination (*n* = 3905)		Second Vaccination (*n* = 3657)	
	N	%	N	%
0	3382	86.6	2551	69.8
1	192	4.9	470	12.9
2	189	4.8	317	8.7
3	66	1.7	117	3.2
4+	62	1.6	109	3.0
Missing	14	0.4	93	2.5
Mean (SD)	0.31 (1.14)		0.62 (1.67)	
Median (IQR)	0 [0]		0 [0,1]	
Any sick leave	509	13.1	1013	28.4

**Table 6:** Association between type of vaccination and proportion of subjects with at least one sick leave day after vaccination, crude, and multiple adjusted risk ratios with 95% confidence intervals.

	N	Sick Leave (*n*)	%	Crude Model		Adjusted Model	
				RR	95%CI	RR	95%CI
First vaccination							
Biontech (Ref)	2982	131	4.4	1.0		1.0	
Moderna	289	38	13.1	3.0	2.1–4.2	2.9	2.0–4.0
Astra	611	336	55.0	12.3	10.2–14.7	11.6	9.7–14.0
Second vaccination							
Biontech (Ref)	2957	777	26.3	1.0		1.0	
Moderna	262	163	62.2	2.4	2.1–2.7	2.0	1.8–2.3
Astra	336	71	21.1	0.8	0.6–1.0	0.7	0.6–0.9

Legend: sick leave (*n*): number of people who had at least one day of sick leave; Ref: reference group; the adjusted model includes age (continuous), sex and profession; nine subjects self-identified as diverse were excluded from first and second vaccination leaving 3896 and 3657 people included, respectively; sick leave was missing among 14 out of 3896 people and 102 out of 3657 people.

## Data Availability

Data is available upon request from: andre.nohl@uk-essen.de.
